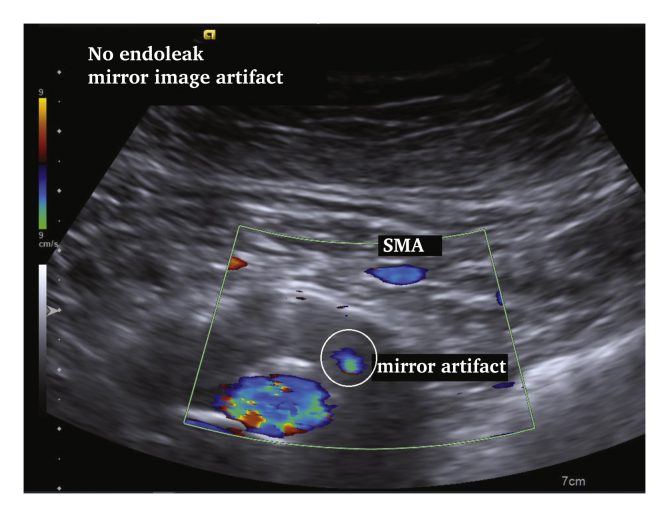# Mirror, Mirror on the Wall'?

**DOI:** 10.1016/j.ejvsvf.2020.11.006

**Published:** 2020-12-13

**Authors:** Jan van Schaik, Rob C. van Wissen

**Affiliations:** Department of Vascular Surgery, Leiden University Medical Centre, Leiden, The Netherlands

Duplex ultrasound (DUS) is increasingly important in the follow up of endovascular aneurysm repair. The “mirror artefact” is an ultrasound image artefact produced when a structure is situated above a strong reflective surface. A duplicate image of the structure is produced behind the “mirror”. A mirror artefact is identified by it being an exact copy of a more superficial structure, despite changing the angle of the transducer. In this DUS follow up case after EVAR (ACUSON S2000 System, HELIX Evolution; Siemens Medical Solutions, Issaquah, WA, USA [Siemens 4C1 1–4MHz convex transducer]), the calcified aneurysm wall led to a mirror image of the superior mesenteric artery, creating the illusion of an endoleak. Recognising ultrasound image artefacts is important to avoid misdiagnosis.

**Figure:**
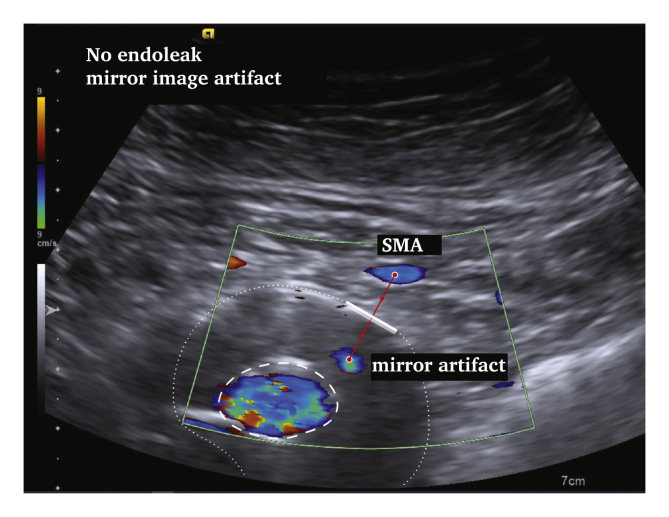

**Figure:**
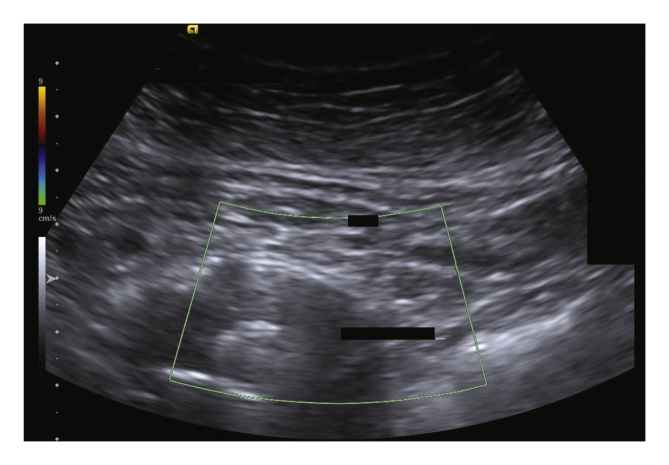

**Figure:**